# The appropriateness of DUNDRUM-3 and DUNDRUM-4 for Māori in forensic mental health services in New Zealand: participatory action research

**DOI:** 10.1186/s12888-020-2468-x

**Published:** 2020-02-11

**Authors:** Julie Wharewera-Mika, Erana Cooper, Nick Wiki, Kiri Prentice, Trudie Field, James Cavney, David Kaire, Brian McKenna

**Affiliations:** 1 Auckland University of Technology, Auckland, New Zealand; 2 Auckland Regional Forensic Psychiatry Services, Waitemata District Health Board, Auckland, New Zealand; 3 Centre for Forensic Behavioural Science, Swinburne University of Technology, Melbourne, Victoria Australia

**Keywords:** Structured clinical judgement, DUNDRUM toolkit, Māori, Forensic mental health services

## Abstract

**Background:**

The Auckland Regional Forensic Psychiatry Services (ARFPS) in New Zealand has introduced structured clinical judgment instruments developed in Ireland (DUNDRUM-3 and DUNDRUM-4) to assist staff decision-making regarding service users’ clinical pathways. In New Zealand, Māori (the indigenous people) constitute 43% of the in-patient forensic mental health population. The aim of this study was to determine the face validity of the measures for Māori.

**Methods:**

Participatory Action Research was aligned with a kaupapa Māori (Māori-orientated) research approach, to give full recognition to Māori cultural values. Two hui (gatherings) were held with Māori clinical and cultural experts at the service. The first hui (*n* = 12), explored the cultural appropriateness of the measures. The second (*n* = 10) involved a reflection on appropriate adaptions to the measures. Discussions were digitally recorded, transcribed and thematically analysed.

**Results:**

Although the usefulness of the measures in enhancing the overall quality of clinical decision-making was confirmed, the DUNDRUM measures were considered to be limited in their ability to fully measure Māori service user progress and recovery. Suggestions were made to develop an additional ‘pillar’ focused on cultural identity and spirituality for DUNDRUM-3; to use both service user and family ratings for the adapted DUNDRUM-3 and DUNDRUM-4 measures; and to involve cultural expertise at the point of structured clinical judgement when using the measures.

**Conclusions:**

This is the first study to consider the face validity of the DUNDRUM-3 and DUNDRUM-4 for indigenous peoples, who are internationally over-represented in forensic mental health services. Suggested changes would require a negotiated, collaborative process between Māori cultural expertise and the original authors of the measures.

## Background

Forensic mental health services internationally are coming under increased pressure to manage burgeoning caseloads within existing resources and infrastructures [1]. The Auckland Regional Forensic Psychiatry Service (ARFPS) in New Zealand has refined its integrated recovery pathways for service users through the service [2]. Clinical decisions regarding service user progress through security pathways has been traditionally based on unstructured, idiosyncratic formulation of security appropriateness by the receiving unit [3]. Within the refined recovery pathways, progress is now determined by attaining key recovery ‘milestones’, which signal success of therapeutic engagement at one level of security to the extent that the service user has demonstrated the ability to manage risks at a reduced level. The three milestones are readiness for entry to the recovery pathway after admission; readiness for the movement from medium to minimum security; and readiness to exit inpatient services and return to supported community living [2].

Entry to the pathway at milestone one is determined by a comprehensive holistic needs assessment and decision-making by a multi-disciplinary panel, independent of the daily management of the service user [2]. The needs identified are used to develop the service user’s holistic care and treatment plan. This plan is aimed at reducing risk and facilitating the recovery journey necessary for eventual discharge and transition to prosocial life in the community [4]. As the service user progresses through the in-patient settings, placement is based on meeting mental health, offending, psycho-social and socio-cultural needs, which are cognisant of risk. As each milestone is achieved the level of security is reduced [5].

To assist in decision making regarding milestone achievement, the service has introduced metrics to inform structured clinical judgment. Such metrics serve to enhance the quality of clinical decision-making by improving consistency and reliability, ensuring research-based items are considered, and enabling a transparent decision-making process with less chance of error [4].

The ARFPS has introduced the clinicians’ version of the DUNDRUM-3 and DUNDRUM-4 as validated, reliable, structured clinical judgment measures to assist staff decisions on milestone achievement. These assist in determining service user functioning, recovery and risk in decision-making for service users to progress through security levels [6, 7]. During the recovery pathway, there is an expectation of completing recovery programmes relevant to holistic needs. DUNDRUM-3 (Programme Completion Scale) measures the completion and learning gained from involvement in such programmes. The programmes relate to seven, theoretically-based ‘pillars’ of care and treatment: (i) physical health, (ii) mental health, (iii) drug and alcohol recovery, (iv) problem (offence related) behaviours, (v) self-care and activities of daily living, (vi) education, occupation and creativity (vocation), and (vii) family and intimate relationships [4]. DUNDRUM-3 allows ratings of each of these, as to the extent to which the person has successfully completed recovery programmes for progression to the next security level [6].

Recovery focuses on the unique personal growth of mental health service users, developed through the lived experience of wellness and illness. Recovery involves the person connecting with those who can support them, having a sense of hope for a life worth living, and building a sense of identity that is socially integrated [8]. DUNDRUM-4 (the Recovery Scale) focuses on seven characteristics that signal progress on the recovery journey; stability, insight, rapport with those who can assist, leave achievement, addressing dynamic risk factors, victim sensitivity and hope. DUNDRUM-4 also allows clinicians to rate each of the characteristics, as to the extent to which the person has sufficiently progressed in their recovery toward the next security level [4].

The clinician rated DUNDRUM-3 and DUNDRUM-4 have been shown to have excellent internal consistency and inter-rater reliability, and predictive ability of conditional discharge by the statutory review board in Ireland [6, 7], and are used in some forensic mental health services in the United Kingdom, Australia and New Zealand [9]. Indigenous peoples are over-represented internationally in forensic mental health services [10]. In New Zealand, Māori (the indigenous people) constitute 15% of the population, yet are 43% of the forensic mental health population [11]. The employment of risk assessment instruments with indigenous people, when not validated with such populations, has been challenged [12]. Therefore, the aim of this research study was to determine the face validity and appropriateness of the use of DUNDRUM-3 and DUNDRUM-4 for Māori within forensic mental health services in New Zealand.

## Methods

### Research design

This research involved exploring the strengths and limitations of the DUNDRUM-3 and DUNDRUM-4 and how they could potentially be enhanced or adapted for future use with Māori tāngata whai i te ora (service users) in forensic mental health services. Given the exploratory nature of this research, a qualitative research approach was undertaken using Participatory Action Research (PAR), situated within a Kaupapa Māori Research (KMR) framework.

PAR values a focus on identifying and addressing an issue, which concerns the participating community. The issue is identified and defined through reflection by the participating community. Addressing the issue then typically involves a cycle of planning (consideration of courses of action), selecting and taking action, and observation and reflection on the consequences and impacts of the actions [13, 14].

KMR stems from a Māori worldview giving full recognition to Māori cultural values and systems, and is carried out according to Māori cultural ethics [15], whereby the issue of research interest are identified by Māori and the outcome of the research considered of benefit to Māori. KMR research is inherently connected to Māori values and aspirations.

The inclusive orientation of PAR aligns particularly well with KMR in that issues and outcomes, which are important to Māori, can take centre stage. PAR provides for research processes of reflection and transformation to occur within a Māori context. It aims to be empowering through building knowledge and solutions that positively impact on those involved in a culturally appropriate and relevant way [14, 16–18]. It is notable that “Both of these research approaches are grounded on research questions generated by the participating community; the answers to which should be emancipatory and result in change” [19].

### Setting

The Auckland Regional Forensic Psychiatry Service (ARFPS) is a 110 bed in-patient forensic mental health secure hospital, providing medium to low security care and treatment integrated on a single campus. The hospital is one of five regional forensic mental health services in New Zealand and serves a catchment population of 1.5 million people. The hospital is organised into a series of eight units from medium acute care to a low secure hostel assisting in the transfer of service users into supported community living options.

In 2015, the ARFPS re-orientated its service delivery by developing three alternative service pathways to recovery developed in relation to patterns of offending, gender sensitivity and cultural need. These are a mixed gender pathway, a male only pathway and a Kaupapa Māori (Māori specific) pathway to recovery [2]. DUNDRUM-3 and DUNDRUM-4 were introduced to inform structured clinical judgment on the achievement of security levels along all pathways.

### Procedure

Māori staff at ARFPS generated this project in response to the service-wide introduction of the DUNDRUM for use with all service users in 2015. The project was led by Māori researchers (with clinical expertise), who contributed to the design and facilitated the data collection and analysis process. A rangahau whānau (research advisory group) was developed which included the researchers, experienced Māori clinicians, a kaumatua (elder) from ARFPS, academic support to the service and a non-Māori clinical lead. This group met together on numerous occasions to discuss and progress the research.

Two one-day hui (gatherings) were held in late 2016 and early 2017 and were facilitated in accordance with Māori tikanga (culturally correct ways of doing things). The purpose of the first hui was to gather initial feedback in relation to the value of the DUNDRUM measures (strengths, limitations, and appropriateness for Māori), and to develop potential actions based on these reflections. The purpose of the second hui was to reflect on the developments made following recommendations for action from the first hui. The researchers facilitated both hui, which were attended by the rangahau whānau (some of whom also took part as participants) and other participants (see below for full information).

Following culturally-specific formalities, the first hui commenced with a discussion about the dual roles of hui participants who were also members of the rangahau whānau. The rangahau whānau comprised of some members of staff who had initiated this study, and some held additional knowledge about the implementation of the DUNDRUM. These staff wished to fully participate in the hui with the other participants. This was considered appropriate. A focus on providing a safe process for all participants to contribute was highlighted, with an emphasis placed on the importance of getting everyone’s thoughts, feedback, and ideas about the DUNDRUM measures.

During this first hui, presentations were made by three members of the rangahau whānau. These presentations were designed to stimulate in-depth discussion. Content included in these presentations was:

1. ‘Te Ao Māori: Part 1’ provided a general overview of the Māori worldview including specific cultural practices relevant to Māori cultural ways of being.
2. The DUNDRUM overview covered the development of the measures (internationally) and a review of the measures themselves, as well as information about their implementation at ARFPS.
3. ‘Te Ao Māori: Part 2’ provided a more in-depth presentation about Māori ways of being and the Māori worldview.
4. An overview was given of the culturally specific pathway in the service. The developmental history of the pathway and its core values were reviewed.

These presentations formed the basis of discussions, which aimed to gather the participants’ perceptions of the value of DUNDRUM (its strengths, limitations, and appropriateness for Māori), with particular regard to developing potential actions based on these reflections.

The second hui was held 2 months later, with the same participants, to reflect on the developments made following the recommendations for action from the first hui. The same two researchers facilitated this hui.

### Participants

Seventeen people attended the first hui. The proceedings were overseen by the kaumatua (who also participated in the hui) and facilitated by the two Māori researchers (both clinical psychologists). All participants in the hui (*n* = 12) were Māori and employed by the ARPFS; four were taurawhiri (cultural workers -two males and two females), three were registered nurses (two males and one female), one was a psychiatric registrar (female), a social worker, a tāngata whai i te ora expert (Māori consumer advisor with lived experience of mental illness), a health care assistant and the kaumatua (all males). All except the registrar and the kaumatua had more than 5 years of experience working in the service, while all except the kaumatua and the health care assistant had experience in the use of the DUNDRUM measures. Three attendees were non-Māori staff and were present in a consulting capacity on points of clarification regarding the DUNDRUM. These were a psychiatrist, a psychologist and a researcher.

A total of 14 people from the first hui attended the reflective hui. The actual participants in the hui (*n* = 10) included taurawhiri, registered nurses, a psychiatric registrar, a health care assistant and the kaumatua. Feedback was obtained later from the tāngata whai i te ora expert, who had not been able to attend the reflective hui. The hui was facilitated by the Māori researchers, and was also attended by the non-Māori psychiatrist and researcher for consulting purposes.

### Data collection and analysis

PAR and KMR approaches to research can involve a variety of quantitative and/or qualitative methods, selected according to the specific topic in question [14]. For this particular study, a wholly qualitative approach was taken. The discussions for the two hui were digitally recorded and written notes taken by the research facilitators. Each topic of discussion was facilitated during the hui until a point of consensus was reached by the participants. These points of consensus involved identifying key areas of concern and actions required in relation to them. Recordings of the two hui were transcribed and then analysed by the Māori researchers, who examined salient themes. Thematic analysis of the transcripts was carried out following the six phases in conducting thematic analysis [20]: becoming familiar with the data, generating initial codes, searching for themes, reviewing themes, defining and naming themes, and producing the report. Examples of the words of participants that supported the themes are highlighted in italics in this manuscript. The data analysis from the first hui was presented back to participants at the second hui and informed the discussions that followed.

## Results

The results from the first hui were drawn from the three areas of discussion posed: (1) the strengths and usefulness of the DUNDRUM-3 and DUNDRUM-4 for tāngata whai i te ora, (2) the limitations of the DUNDRUM measures for tāngata whai i te ora, and (3) recommendations for the adaption of the DUNDRUM measures to be more suitable for tāngata whai i te ora. Within each of these three discussion areas, key themes were identified and are reported below.

### Strengths of the existing measures

#### Robustness and clinical utility

Participants were impressed by the rigor applied in developing the DUNDRUM measures, seeing them as:

“*highly validated and reliable measures*”.

Their use was seen as an improvement on the traditional reliance on unstructured clinical judgment or on developing alternative service-specific measures from scratch:

“*We have access to this tool that has already been widely tested and offers many benefits for us – so why wouldn’t we use it?*”

Many participants saw the value in the use of DUNDRUM-3 & DUNDRUM-4 because they aligned recovery needs with specific programmes or interventions. The measures were seen as having pragmatic clinical utility, in determining the achievements required in order for tāngata whai i te ora to progress on their pathway through the security levels of the service. This enabled a recovery journey, which was systematic and transparent:

*“They* [tāngata whai i te ora] *can move, progress through the service, because of what they’re achieving. The actual healthcare process is more meaningful, because we understand what someone’s got to do to move forward. For example, if you want to deal with your violence, you have to attend violence prevention*”.

#### Public safety

The strengths of the measurements where seen as extending beyond the mapping of tāngata whai i te ora progress and recovery, to assisting in decision-making regarding wider obligations to public safety. The use of the measures gave a clear indication of what was required for tāngata whai i te ora readiness to move to the next (lower) level of security toward eventual discharge and safe reintegration with whānau (family) and the community. As stated by one participant:

“[the use of the DUNDRUM] *tracks change, in a way measuring risk and creating safer communities by decreasing the potential for violence”.*

### Limitations of the measures for tāngata whai i te ora

#### Cultural deficits

Despite the benefits of DUNDRUM-3 and DUNDRUM-4, participants expressed concerns about cultural deficits in the use of the measures in determining the holistic wellbeing of Māori. Participants recognised that the DUNDRUM was not created in New Zealand and therefore was not expected to include cultural elements specifically relevant to Māori. Overall, participants considered the most prominent limitation of the measures was their failure to capture the essence of what it is to be Māori, as a reflection of Te Ao Māori (cultural principles, values and beliefs) and cultural expressions of this world view:

“*What you’ll find is that there are a number of areas that are missing for us, it’s obviously wairuatanga* (spirituality) *is first and foremost, it’s the most important. The importance of tikanga* (the correct culturally specific way of going about things) *and kawa* (protocols) *as a point of difference, that controls everything; it controls how you use the service … ..*”.

#### Use of the measure in decision making

A discussion amongst participants indicated a concern about lack of consistency regarding the use of the DUNDRUM measures at the point of exercising clinical judgement:

*“But whether that (clinical judgement) happens practically in this service or not is another matter*”.

This concern stemmed from the use of measures only undertaken by clinicians. A clinical perspective alone was seen as conflicting with an inclusive recovery approach, as there were limited opportunities for the perspective of tāngata whai i te ora and their whānau (family) to be considered. As stated:

“*Who are the milestones determined by? Is there the opportunity for the DUNDRUM to be used more collaboratively with tāngata whai i te ora*”.

### Options for adaptation

Given the benefits and limitations of the DUNDRUM, participants strongly felt it would be useful to explore options to adapt the measures to ensure suitability of use with tāngata whai i te ora. Themes articulated as options were: (1) the inclusion of Te Ao Māori (cultural principles, values and beliefs) throughout all of the existing DUNDRUM-3 and DUNDRUM-4 measures, (2) the addition of a new ‘pillar’ in DUNDRUM-3 to capture the essence of being Māori, and (3) incorporating a Māori approach in the process of undertaking the DUNDRUM measures.

#### The inclusion of Te Ao Māori throughout existing

Participants agreed the inclusion of core Māori concepts must be included somewhere in the DUNDRUM measures:

“*What is missing, and what’s important to us as Māori, that needs to be factored in*”.

Participants identified specific ‘pillars’ of the DUNDRUM-3 and recovery items in DUNDRUM-4, which already align with aspects of Te Ao Māori. These were seen as a potential starting point for integrating core cultural values throughout the measures:

*“This provides us with clear stages of where to start and where we’re going”.*

Furthermore, the specific core Māori values to be integrated in the DUNDRUM measures were highlighted:

“*Drawing from some of the kupu* (words) *that have been discussed today, about some of the gaps … I think wairuatanga, tikanga came up, whanaungatanga* (sense of family connection)*, cultural identity, level of mātauranga* (cultural knowledge)”.

These key values and concepts were seen as encompassing a broader holistic perspective for tāngata whai i te ora wellbeing, but it was reiterated that it must be Māori defining this integration, as only they hold the knowledge of Te Ao Māori. In this integration, some participants reported the significance of the use of Māori language, given the depth of meaning often lost in translation into English:

“*The importance of language, Māori kupu can help explain what this process is about, it’s important to capture this model using our language”.*

#### The addition of a new “pillar’ to DUNDRUM-3

An alternative to integration was also expressed as a pervading theme. There was a feeling that Te Ao Māori might be more effectively captured in DUNDRUM-3 by the development of a separate ‘pillar’ that stood alongside the existing seven pillars. As stated:

“*We need our own Pou* (pillar)*, we don’t like things just slid in. I’m for the separate Pou to keep our kaupapa Māori ways safe.”*

In this regard, there was a view that there was danger of diluting cultural values through integration. Acknowledging these values in their own distinct domain was seen as a way of protecting Te Ao Māori:

*“So, the idea of the Pou was to protect, to keep everything together. The idea of, for now, DUNDRUM using our Pou once it’s developed”.*

There was also a more pragmatic concern that integration might compromise the validity of the existing measures. A separate ‘pillar’ was seen as the least disruption to measures which were primarily valued:

*“My preference is to add something that makes it safe, and does not takahi (stamp) on the DUNDRUM work, we want to add something to make it better, something we’re happy does a good job”.*

There was general satisfaction with the recovery measure (DUNDRUM-4) remaining as it exists.

#### Incorporating a Māori approach in undertaking the measures

The participants also considered the process of undertaking the measures as a means of enhancing the measures themselves. It was culturally incomprehensible that the measurement of recovery should only occur from the clinician’s perspective. There was a strong expression that the measures should incorporate not only the perception of tāngata whai i te ora, but of their whānau (the wider kinship-based entities they belong to):

“*If we were gonna move down the path of measures, we need self-assessment, where tāngata whai i te ora sit down and do this questionnaire; the clinician does it; and the whānau do it. The level is that between the three of us, we can negotiate and work on a way forward”.*

Participants also stated that Māori cultural experts employed by the service (Taurawhiri) should be involved when the measures are used to inform structured clinical decision-making. The measures alone would not unravel the nuances of cultural issues that might require further clarification. The decision-making process was seen as requiring cultural competency.

“People … *competent in that mātauranga* [Māori knowledge]*”.*

### Action

At the end of the hui, the majority of participants agreed further discussion would be required. It was agreed by participants that a smaller steering group (a sub-group of the participants) be established to clarify the three options that evolved in the gathering, with the view of returning to the wider group of participants for further consultation and endorsement. The following quote clarifies this process:

*“We think collectively, as a people, and we work better together as well. And it’s about inclusiveness. But I also understand that there are benefits when we work with a smaller rōpū* (group) *as well. You can push through and get things done quicker”.*

The kaumatua (elder) facilitating the hui cautioned not to rush this process and rather take time in the decision-making:

“[I suggest] *taking our time and not being rushed by others’ agendas*”.

### The follow-up hui

The participants in the second follow-up hui reinforced their support for the outcomes of the initial hui, which they felt had provided a solid foundation to develop the DUNDRUM measures to be more responsive for tāngata whai i te ora. The steering group, which included 3 Maori clinicians and the kaumatua, presented their review of the ‘3 ‘potential’ actions for developing kaupapa Māori enhanced DUNDRUM measures. The landing point was the development of a separate ‘pillar’ for Te Ao Māori, which was seen as the least disruption to the measurements that had been rigorously developed to date. As stated:

*“The bones are there, so don’t tamper with it, be careful we’re not adding too much that it’s being corrupted”.*

The majority of participants agreed to the addition of a new ‘pillar’ on “Culture and Spirituality”’ to the existing DUNDRUM-3 would be the appropriate way to enhance the measures for tāngata whai i te ora. The hui also supported an enhanced process in undertaking the measures, to include tāngata whai i te ora self- assessment and whānau assessment into both DUNDRUM-3 and DUNDRUM-4. This was seen as an extension of the development of the addition ‘pillar’ to DUNDRUM 3. They all supported the involvement of cultural expertise at the time of using the measures to assist clinical decision-making.

### Action

The steering group, supported by all participants, was tasked with ensuring momentum of the project developments continued. It was suggested that more cultural input might be helpful. To this end, the steering group presented the project and its outcomes to the kaumātua (elders) reference group of the health authority the ARFPS is responsible to. The group supported the process and outcomes in principle, but requested further consultation once the ‘pillar’ had been developed.

## Discussion

Risk assessment tools in forensic mental health settings rarely include validation with indigenous and ethnically diverse populations [12]. In Canada, the Supreme Court has held that the Correctional Service of Canada breached its statutory duty to an inmate of indigenous descent, in assessing his risk of recidivism using actuarial risk assessment tools that had not been proven to be accurate when applied to indigenous people [21]. This finding opens the challenge to processes not validated with indigenous and ethnically diverse people, including the use of structured clinical judgment tools.

This study was aimed at investigating the face validity of a structured clinical judgment tool to assist in determining the pathway of service users through a forensic mental health service, where the majority of the population are of indigenous descent [11]. In determining the face validity of the DUNDRUM-3 and DUNDRUM-4, Māori believed the measures helped to determine which programmes needed to be achieved to assist the progress of the recovery journey of Māori service users (tāngata whai i te ora). However, for the measures to be safely used with Māori, some crucial adaptations are required: The addition of another ‘pillar’ to DUNDRUM-3 focusing on the cultural and spiritual essence of what it is be Māori, the inclusion of a service user and family perspective in the use of both the Dundrum-3 and DUNDRUM-4 measures, and the use of cultural expertise when the measures are actually used to inform clinical judgment.

Overall, participants considered the most prominent limitation of the DUNDRUM measures was the failure to capture the essence of what it is to be Māori as a reflection of Te Ao Māori. Te Ao Māori refers to a specific cultural world view involving principles, values and beliefs. This way of life requires specific cultural practices (tikanga), that provide protection and acknowledgement of the world view [22].

Central to this world view (in the words used by participants in this study) is the concept of wairuatanga, simplistically translated as spirituality. Yet wairuatanga embraces “the principle of cultural integration that hold all things together over time; it is as material as it is metaphysical ; as contemporary as it is ancestral” ([23], p 87). It is not confined to the individualistic notion of personal spiritual growth implied by “self actualisation”, but rather self-transcendence, whereby individualistic benefit is subsumed within commitment to the common good achieved by serving others [24].

This goal and the associated practices are expressed as a cultural need for tāngata whai i te ora in their pathway through the ARFPS. Learning and support for tāngata whai i te ora presently occurs to achieve this need. Yet the present version of the DUNDRUM-3 does not capture this.

In this study, adaptation was projected to achieve the goal of incorporating spirituality and cultural identity with as little disruption as possible to the existing DUNDRUM-3 and DUNDRUM-4, given that they have been validated [6]. Therefore rather than integrating key cultural concepts into the existing ‘pillars’ of DUNDRUM-3, an additional separate ‘pillar’ covering cultural identity and spirituality was suggested. In discussing the theory of item ratings of DUNDRUM-3 and DUNDRUM-4, Kennedy et al. [4] identify the influence of Maslow’s hierarchy, the trans-theoretical model of stages of change, and the five stages of recovery. In the table associated with this discussion, “spiritual and cultural integration” is eluded to but not discussed ([4], p.50). There is the suggestion that “spiritual and cultural integration” involves ratings on a continuum from alienation, to “trading” interactions, to accepting and committing to “communal and social customs and affiliations”, to accepting and committing to “communal and social customs of value and virtue”, to “self -transcendence” for “communal good”. This mention and acknowledgement provide a basis for discussions on the development of a separate spiritual and cultural “pillar”.

In a review of routine outcome measures for forensic mental health services, Shinkfield and Ogloff [25] highlighted that the DUNDRUM toolkit fell short of assisting the assessment of recovery by not providing a service user perspective in the measures. This has been rectified by the introduction of service user self-assessment validated in both DUNDRUM-3 and DUNDRUM-4 [26]. Concern in this study for the lack of both service user and family perspectives in the measures appears in some part to be related to the implementation of an older version of the DUNDRUM toolkit and the failure of the service to update this. However, the self-rating service user version of DUNDRUM would require consideration of the additional pillar, and also a family version of both the DUNDRUM-3 and DUNDRUM-4 developed. The family perspective is not a dimension considered to date.

The use of the measurements should be of “assistance when making decisions about evidence of change and readiness for a move to less secure or community settings” [4] and not a definitive determination of such decisions. The addition of a cultural dimension would require cultural expertise to clarify cultural complexity at the point of decision making. The employment of cultural expertise (taurawhiri) at the ARFPS already provides such a mechanism.

The participants in this study were mindful of avoiding ‘trampling” on the considerable work undertaken by those involved in developing the DUNDRUM measures. The development of an additional pillar on both the service user and clinical versions of DUNDRUM-3, and a family version of DUNDRUM-3 and DUNDRUM-4 would require collaboration with the authors of the existing measures, if they so consented. It would also need to involve the inclusion of those with clinical, lived experience and family expertise. Whether this was a pillar to target Māori or the development of a transcultural pillar would be part of this negotiation. The outcome would then need to be psychometrically tested for validity and reliability in the jurisdiction(s) targeted. Once validated in English, further thought would need to be given to the need for rigorous linguistic and cultural translation into Te Reo (the Māori language) [27].

### Limitations

This participatory action research is limited by data collected from Māori staff in one regional forensic mental health service, albeit a service with a majority of service users who are Māori. There was limited involvement of those with lived experience or whānau (family) expertise. The analysis relied on a small number of participants some of whom were unable to attend the follow up gathering. As such, data may not represent the perceptions of Māori staff in other forensic mental health services throughout Aotearoa- New Zealand. Furthermore, data was recorded from the total gatherings, which negated the ability to attribute supporting data to individual participants, despite nearly all participants having both clinical and cultural expertise. Finally, no consideration was given to cultural weighting for the admission criteria reflected in the use of DUNDRUM-1 and DUNDRUM-2. If cultural modification of DUNDRUM-3 and DUNDRUM-4 were to proceed, then this would be a worthy consideration.

## Conclusion

This is the first study of its kind to consider the face validity of the DUNDRUM-3 and DUNDRUM-4 with indigenous peoples, who are internationally over-represented in forensic mental health services [10]. Although this participatory research confirmed the usefulness of the DUNDRUM-3 and DUNDRUM-4 in enhancing the overall quality of clinical decision-making, the measures were considered to be limited in their ability to fully measure tāngata whai i te ora progress and recovery, in a way that was culturally relevant, meaningful, and useful. Suggestions were made to improve the DUNDRUM-3 and DUNDRUM-4 in this regard: The development of an additional cultural identity and spirituality ‘pillar’ in DUNDRUM-3; the involvement of both service user and family ratings of an adapted DUNDRUM-3 and DUNDRUM-4; and the involvement of cultural expertise at the point of structured clinical judgement when using the measures. Such changes would require a negotiated, collaborative process between Māori expertise and the original authors of the measures.

## Data Availability

The datasets used during the current study are available for the corresponding author on reasonable request.

## Abbreviations

ARFPS: Auckland Regional Forensic Psychiatry Services
DUNDRUM-3: Programme Completion Scale
DUNDRUM-4: Recovery Scale
Hui: Gathering
Kaumatua: Elder
Kaumātua: Elders
Kaupapa Māori: Māori-orientated
Kawa: Protocols
KMR: Kaupapa Māori Research
Kupu: Words
Māori: Indigenous people of New Zealand
Mātauranga: Knowledge
PAR: Participatory Action Research
Pou: Pillar
Rangahau whānau: Research advisory group
Rōpū: Group
Tāngata whai i te ora: Māori service users
Taurawhiri: Cultural workers
Te Ao Māori: Māori worldview (cultural principles, values and beliefs)
Te Reo: The Māori language
Tikanga: The culturally correct ways of doing things
Wairuatanga: Spirituality
Whānau: Extended family
Whanaungatanga: Sense of family connection
